# Residential Proximity to Roadways and Ischemic Placental Disease in a Cape Cod Family Health Study

**DOI:** 10.3390/ijerph14070682

**Published:** 2017-06-24

**Authors:** Amelia K. Wesselink, Jenny L. Carwile, María Patricia Fabian, Michael R. Winter, Lindsey J. Butler, Shruthi Mahalingaiah, Ann Aschengrau

**Affiliations:** 1 Department of Epidemiology, Boston University School of Public Health, Boston, MA 02118, USA; jennycarwile@gmail.com (J.L.C.); aaschen@bu.edu (A.A.); 2 Department of Environmental Health, Boston University School of Public Health, Boston, MA 02118, USA; pfabian@bu.edu (M.P.F.); ljbutler@bu.edu (L.J.B.); 3 Data Coordinating Center, Boston University School of Public Health, Boston, MA 02118, USA; mwinter@bu.edu; 4 Department of Obstetrics & Gynecology, Boston University School of Medicine, Boston, MA 02118, USA; shruthi@bu.edu

**Keywords:** ischemic placental disease, placenta, pregnancy, air pollution, traffic

## Abstract

Exposure to air pollution may adversely impact placental function through a variety of mechanisms; however, epidemiologic studies have found mixed results. We examined the association between traffic exposure and placental-related obstetric conditions in a retrospective cohort study on Cape Cod, MA, USA. We assessed exposure to traffic using proximity metrics (distance of residence to major roadways and length of major roadways within a buffer around the residence). The outcomes included self-reported ischemic placental disease (the presence of at least one of the following conditions: preeclampsia, placental abruption, small-for-gestational-age), stillbirth, and vaginal bleeding. We used log-binomial regression models to estimate risk ratios (RR) and 95% confidence intervals (CI), adjusting for potential confounders. We found no substantial association between traffic exposure and ischemic placental disease, small-for-gestational-age, preeclampsia, or vaginal bleeding. We found some evidence of an increased risk of stillbirth and placental abruption among women living the closest to major roadways (RRs comparing living <100 m vs. ≥200 m = 1.75 (95% CI: 0.82–3.76) and 1.71 (95% CI: 0.56–5.23), respectively). This study provides some support for the hypothesis that air pollution exposure adversely affects the risk of placental abruption and stillbirth; however, the results were imprecise due to the small number of cases, and may be impacted by non-differential exposure misclassification and selection bias.

## 1. Introduction

Optimal placental vascularization is important for a healthy pregnancy, as the placenta is responsible for the fetal-maternal exchange of gas and nutrients [1]. Preeclampsia, placental abruption, and intrauterine growth restriction are pregnancy conditions that may share a common pathophysiologic mechanism through placental insufficiency, due to the incomplete trophoblast invasion of the maternal spiral arteries [2]. Placental insufficiency causes uteroplacental underperfusion, chronic hypoxia, and placental ischemia [3,4], and can lead to serious maternal and fetal complications including hemorrhaging, maternal mortality, fetal distress, brain damage, preterm delivery, and stillbirth [2,5,6,7]. Given the common mechanistic origins and epidemiologic patterns of preeclampsia, placental abruption, and intrauterine growth restriction, the term ischemic placental disease has been coined to describe a pregnancy affected by at least one of these conditions [4]. Some risk factors for ischemic placental disease have been identified (for example, an older maternal age, non-white race, and lower socioeconomic status) [8], but many risk factors remain unknown.

Environmental exposures such as air pollution may impact placental function through several mechanisms, including systemic inflammation and oxidative stress, endothelial dysfunction, decreased DNA methylation, disturbances to hemodynamic responses, and systemic changes in hematocrit and blood viscosity [1,9,10,11,12,13]. Motor vehicles are a major source of ambient air pollution [14], and the recent growth of the world’s motor vehicle fleet has resulted in a larger proportion of the population living close to busy highways and roads [14]. These individuals are potentially exposed to higher levels of tailpipe emissions (which contain carbon monoxide, carbon dioxide, hydrocarbons, nitrogen dioxide, particulate matter, and mobile source air toxics such as benzene), non-emission exposures such as re-suspended road dust and particles from brake and tire wear, and noise [14].

There is a substantial literature assessing the association between the individual components (preeclampsia, placental abruption, and intrauterine growth restriction) of ischemic placental disease and air pollution exposures. Prior studies have found evidence of a positive association between traffic and the risk of preeclampsia [15,16,17,18] and intrauterine growth restriction, in addition to other adverse pregnancy outcomes [19,20,21,22,23]; however, some studies have found no association [17,24,25,26,27]. Studies exhibit substantial heterogeneity, particularly in exposure assessment methods. Many studies assessing the relationship between air pollution exposure and birth outcomes rely on data from air quality monitoring networks [28], but these methods can only be used in areas in which sufficient monitoring data are available. Road proximity and traffic density metrics are straightforward, widely available measures that can capture long-term local variation in exposure to the mixture of pollutants that comprise traffic-related air pollution [29]. In the present analysis, we use these metrics in a Massachusetts-based retrospective cohort study to examine the association between exposure to traffic-related air pollution and the risk of ischemic placental disease and other obstetric conditions with a placental etiology.

## 2. Materials and Methods

### 2.1. Study Population

**Figure 1:**
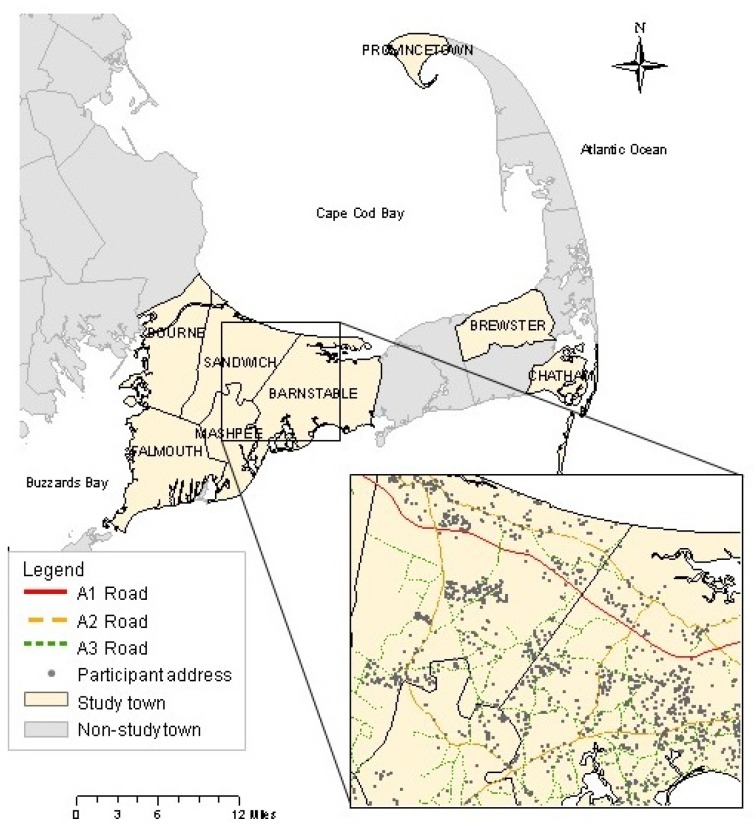
Study area in the Cape Cod region of Massachusetts. The eight study towns are: Barnstable, Bourne, Brewster, Chatham, Falmouth, Mashpee, Provincetown, and Sandwich. Major roadways, including A1, A2, and A3 roads, are shown in the pop-out map, along with the location of participant residential addresses (jittered).

Data used in this analysis were collected for the Cape Cod Family Health Study, a population-based retrospective cohort study initiated in 2002 to examine the effects of exposure to perchloroethylene (PCE) through contaminated drinking water on reproductive and childhood health [30,31,32]. A detailed description of the cohort has been published elsewhere [33]. Briefly, women who gave birth to at least one child (identified here as “index” pregnancies) and resided in one of eight Cape Cod towns (Figure 1) between 1969 and 1983 were eligible for inclusion in the study. From 2002 to 2003, we attempted to obtain the current contact information for eligible mothers and their partners. We sent introductory letters and self-administered questionnaires to all eligible participants who could be traced. We followed-up with non-respondents twice more by mail, and then attempted to contact them by telephone.

The self-administered questionnaire included information on demographics (age, race/ethnicity, education, occupation); medical history; pregnancy history (pregnancy outcome, date pregnancy ended, length of pregnancy, pregnancy complications, parental age, smoking and alcohol consumption by trimester, prenatal care, multivitamin and medication use in pregnancy, gestational weight gain, presence of birth anomalies, and birthweight of child); information on occupational exposures; and residential history. For index pregnancies, additional parental demographic and pregnancy characteristics were available from birth certificates. PCE exposure assessment was based on a leaching and transport algorithm embedded in water flow modeling software, as has been described in detail elsewhere [30].

This study was approved by the institutional review boards at the Massachusetts Department of Public Health, Boston University Medical Center (No. H-32438), and the 24A/B/11B Review Committee at the Massachusetts Department of Public Health.

### 2.2. Exclusions

Study participants reported a total of 6519 pregnancies, including both index and non-index pregnancies. We excluded pregnancies with an unknown outcome (*n* = 14), ectopic pregnancies (*n* = 49), elective abortions (*n* = 367), pregnancy losses at <27 weeks’ gestation (*n* = 389), multiple births (*n* = 55), fetuses with major birth anomalies (*n* = 124), and pregnancies with an unknown date of the last menstrual period (*n* = 178). Of the remaining pregnancies that occurred in one of the eight study towns (*n* = 3653), we additionally excluded pregnancies at addresses that could not be geocoded (*n* = 159) and pregnancies with an incalculable PCE exposure (*n* = 185). The final sample for this analysis was 3309 pregnancies.

### 2.3. Assessment of Ischemic Placental Disease

We defined ischemic placental disease as the presence of at least one of the following conditions in a given pregnancy: preeclampsia, placental abruption, or small-for-gestational-age (SGA). On the questionnaires, women reported the outcome of each of their pregnancies (live birth, stillbirth, miscarriage, induced abortion, or ectopic pregnancy), as well as the date that the pregnancy ended. They also reported the birth weight, gestational age (in categories of <8, 8–13, 14–26, 27–36, ≥37 weeks), and whether each pregnancy was complicated by placental abruption or separation, preeclampsia, or vaginal bleeding. To classify SGA, defined as birth weight <10th percentile of a gestational age- and sex-specific distribution for singleton births [34], we used data on the birth weight (available for all pregnancies) and gestational age from birth certificates (available for index pregnancies only). For non-index pregnancies (*n* = 1385), we used the birth weight from the self-administered questionnaire and multiple imputation to obtain continuous values of gestational age. We used PROC MI (SAS Institute version 9.3) to create five imputed data sets based on a model of 72 variables, including the birth weight and categorical gestational age.

### 2.4. Assessment of Exposure to Traffic-Related Air Pollution

Exposure metrics were derived using proximity analysis, which is a widely used exposure assessment technique; multiple studies have found relationships between proximity-derived air pollution metrics and adverse pregnancy outcomes [16,17,18,35]. To obtain the maternal residential addresses throughout gestation, the mothers reported on the self-administered questionnaire the exact street address, nearest cross street, and calendar years of occupancy for each of their family’s Cape Cod residences from 1969 to 1990. Addresses were geocoded using ArcGIS 8.1 (ESRI, Redlands, CA, USA). Geocoding was conducted without knowledge of pregnancy history or PCE exposure levels. Of the 5324 reported addresses, 87.6% were successfully geocoded to a land parcel; 9.6% were geocoded to the nearest cross street or middle of the street (when the house number was missing); and 2.7% could not be geocoded and were excluded from the analysis.

Road data were obtained from Topologically Integrated Geographic Encoding and Referencing System (TIGER) files for Barnstable County (which includes Cape Cod) from the 1990 U.S. census website for each of the eight study towns [36]. We incorporated these data into a geographic information system along with the town boundaries and Massachusetts Level 3 parcel data. TIGER road locations were compared to the Level 3 parcel maps (generated with orthoimagery) and the road locations were corrected manually for all towns [37]. We selected road segments from the TIGER files by U.S. census feature class codes to include major roadways, defined as A1 (primary highways with limited access including roads like interstate highways), A2 (primary roads without limited access like state and local highways that connect cities and towns), and A3 (smaller secondary roads that may connect smaller towns) road segments (Figure 1). We used ArcGIS to calculate two metrics of traffic exposure: (a) the shortest Euclidean distance between each residence and the closest major roadway and (b) the length of major roadways within 200 and 500 m buffers around each residence. We also examined the shortest Euclidean distance between each residence and the closest highway (A1 and A2 roads) and the closest primary highway (A1 roads), but the number of cases in the high exposure categories were too small to include in the statistical analysis. For pregnancies in which the mother moved during the pregnancy (*n* = 258), we averaged her exposures from each residence. Based on the distribution of distances in our cohort and prior research showing the decay of pollutants around major roadways [38,39], we categorized distance as <50, 50–99, 100–199, and ≥200 m for analysis. We categorized the length of major roadways within each of the three buffers by setting 0 as the reference group (i.e., no major roadways within the buffer) and dichotomizing the length variable above 0.

### 2.5. Statistical Analysis

We used log-binomial regression models to calculate risk ratios (RR) and 95% confidence intervals (CI) for the association between traffic exposure and ischemic placental disease. We modeled the associations using generalized estimating equations to account for correlation between pregnancies contributed by the same mother [40,41]. We selected potential confounders from those available on the self-administered questionnaires and medical records based on a literature review and construction of a directed acyclic graph (Figure S1). We ran bivariate models controlling for one potential confounder at a time. Variables that changed the association between traffic exposure and ischemic placental disease by >5% were included in the final models; only the maternal age (included in the model as a continuous variable) met this criterion. Because the concentration of air pollutants has decreased over time, we also controlled for the year of pregnancy as a continuous variable in all models. We conducted separate analyses for ischemic placental disease (defined above), preeclampsia, placental abruption, and SGA. We also examined the association between traffic exposure and stillbirth and vaginal bleeding, conditions which may result from placental dysfunction. Lastly, we ran models including stillbirth in the definition of ischemic placental disease, as has been done by other researchers [33].

Prior ischemic placental disease is a strong risk factor for subsequent ischemic placental disease, although it is unknown whether this is due to common behaviors and exposures during each pregnancy or genetic predisposition [3,42]. However, if the etiology of prior and current ischemic placental disease is the same, controlling for prior ischemic placental disease could also cause index event bias, which leads to bias towards the null [43]. Therefore, we ran all models with and without the inclusion of prior ischemic placental disease.

We conducted a sensitivity analysis using the first, second, and third address to define exposure for women who moved during pregnancy (*n* = 258), rather than the average. We also conducted analyses restricting the analytic data set to index pregnancies only (*n* = 1924), for whom outcome information was complete (*i.e.*, for whom we did not have to impute data on the continuous gestational age).

We examined the effect modification by parity (parous vs. nulliparous), season of birth (summer vs. not), maternal age (<30 vs. ≥30 years), and first trimester smoking (any vs. none).

## 3. Results

Overall, 1739 women contributed 3309 pregnancies to the analysis; 41.7% of women had two pregnancies and 21.1% had more than two pregnancies. The majority of study participants were white (96.8%) and educated (80.9%, with at least some college education); around one-third of mothers had husbands with blue collar jobs.

At least one ischemic placental disease was present in 270 (8.2%) of the study pregnancies. Preeclampsia, placental abruption, and SGA were present in 0.9%, 1.1%, and 6.5% of pregnancies, respectively. Most of the affected pregnancies (96.3%) were characterized by a single condition. There were 19 stillbirths and 215 reports of vaginal bleeding. Ischemic placental disease was more common among younger mothers with a lower education and whose partner reported a blue collar occupation (Table 1). Nulliparity and prior ischemic placental disease were strongly positively associated with ischemic placental disease in the study pregnancy. Inadequate gestational weight gain and cigarette smoking or alcohol consumption in the first trimester were also more likely in pregnancies affected by ischemic placental disease.

Mothers lived between 12 and 1923 m from the closest A1, A2, or A3 road during their pregnancies, with a median distance of 188 m (Table 2). Four hundred and seventy-eight (14.5%) pregnancies were to mothers who lived within 50 m of a major roadway, whereas 1511 (45.7%) pregnancies were to mothers who lived ≥200 m from a major roadway. Only 4.3% of pregnancies were to mothers who lived <500 m from an A1 road. Women who lived <50 m from the closest major roadway were more likely to be non-white (4.4% vs. 2.4%), less educated (2.1% vs. 0.8% without a high school diploma), and were more likely to have partners working blue collar jobs (35.8% vs. 34.5%) (Table 3). However, these differences were subtle, which attests to the relative homogeneity of the study population. Other pregnancy-related characteristics were similar across exposure groups.

We found no evidence of an association between traffic exposure and ischemic placental disease (Table 4). Compared with pregnancies to mothers living ≥200 m from the closest major roadway, pregnancies to mothers living 100–199, 50–99, and <50 m from the closest major roadway had 0.87 (95% CI: 0.63, 1.21), 1.08 (95% CI: 0.75, 1.55), and 0.74 (95% CI: 0.47, 1.17) times the risk of ischemic placental disease. The length of major roadways within a 200 or 500 m buffer around the residence was not substantially associated with ischemic placental disease (Table 4).

When we examined the individual components of ischemic placental disease and other obstetrical outcomes that may be related to placental dysfunction, we found some evidence that traffic exposure is associated with an increased risk of placental abruption and stillbirth (Table 5; adjusted RR for living <100 vs. ≥200 m from major roadways = 1.75 (95% CI: 0.82, 3.76) and 1.71 (95% CI: 0.56, 5.23), respectively). Due to small numbers, these analyses were underpowered. Neither metric of traffic exposure was associated with increased risks of preeclampsia, SGA, and vaginal bleeding; in fact, we found some evidence of a lower risk of preeclampsia and vaginal bleeding among mothers with the highest levels of traffic exposure.

Restricting the analytic sample to index pregnancies only did not substantially alter the results (compared with pregnancies to mothers living ≥200 m from the closest major roadway, RRs for living 100–199, 50–99 and <50 m from the closest major roadway were 0.80 (95% CI: 0.52, 1.23), 1.07 (95% CI: 0.67, 1.71), and 0.77 (95% CI: 0.44, 1.35), respectively). When we included stillbirth in the definition of ischemic placental disease, 17 pregnancies were re-classified, but the associations with traffic exposure did not change substantially (RR for living <50, 50–99, and 100–199 vs. ≥200 m from the closest major roadway = 0.89 (95% CI: 0.65, 1.23), 1.13 (95% CI: 0.80, 1.61), and 0.73 (95% CI: 0.47, 1.14), respectively). Using the exposure from the first, second, or third address for women who moved during pregnancy, rather than the average exposure, did not substantially affect the results (data not shown). The results were similar when we controlled for prior ischemic placental disease.

We found no evidence of effect measure modification by parity, season of birth, maternal age, or first trimester cigarette smoking for the analysis of traffic exposure and ischemic placental disease and SGA (data not shown). We were unable to stratify models for preeclampsia, placental abruption, and stillbirth due to small numbers.

## 4. Discussion

In this retrospective cohort study, we found no substantial association between traffic exposure and ischemic placental disease overall. However, we found that the risk of specific obstetrical outcomes related to placental dysfunction, mainly placental abruption and stillbirth, may be higher among women who live close to major roadways or who have a higher density of major roadways around their home. Although these results were imprecise, the strength of the point estimates supports the possibility of a true association.

Our results are reasonably consistent with prior ecologic evidence that has demonstrated an association between city- or county-wide air pollution levels and stillbirth rates [44,45,46]. These data are supported by results from some [47,48,49,50], but not all [51], retrospective cohort studies that have assessed the relationship between stillbirth (assessed from birth records) and individual air pollutants at the stationary monitor nearest the mother’s residence. Inconsistency in the literature may result from the heterogeneous nature of stillbirth, which may be due to placental dysfunction, but can also be caused by fetal, uterine, maternal, amniotic fluid, or umbilical cord problems; trauma either related or unrelated to birth; and unknown causes [52]. 

Our results are also consistent with two studies that have measured the effects of air pollution on placental function. In the Generation R study in the Netherlands, prenatal exposure to nitrogen dioxide (NO_2_) and particulate matter (PM_10_) was adversely associated with measures of placental growth and function [53]. In a cross-sectional study of low-risk pregnant women in Brazil, short-term exposure to NO_2_, measured by personal passive samplers, was associated with decreases in several measures of placental vascularization [1]. However, to our knowledge, only one prior study has assessed the association between air pollution and placental abruption. This retrospective cohort study of pregnancies in Japan [25] from 1997 to 2012 found that living <200 m from a major roadway was not associated with the odds of placental abruption or placenta previa, but was associated with increased odds of the preterm premature rupture of membranes, a condition caused by intrauterine inflammation [54]. 

A substantial literature has demonstrated an effect of air pollution exposures on fetal growth through the decreased placental transport of oxygen and nutrients [35,55]. However, studies have found mixed results when using road proximity as a proxy for traffic-related air pollution, with some demonstrating an increased risk of SGA among those living closest to major roadways [19,20,21,22,23], while others show no association [17,24]. Likewise, findings for exposure to traffic and the risk of preeclampsia [15,16,17,25,26,27] are inconsistent. The use of SGA as a marker of intrauterine growth restriction, the complex etiology of preeclampsia, as well as varying definitions and categorizations of traffic exposure, may contribute to the inconsistency in the literature.

Outcome data were self-reported in this analysis, allowing for the possibility of misclassification. Prior research comparing maternal self-report with medical record data indicates that birthweight is reported accurately [56] and gestational age is reported reasonably well [57]. Preeclampsia and placental abruption are reported with high specificity, but potentially low sensitivity [57,58,59]. However, imperfect sensitivity with near perfect specificity generates no bias in estimating the ratio measures of association [60]; therefore, we do not anticipate that outcome misclassification substantially biased our results. In addition, we used SGA as an estimate of intrauterine growth restriction, a measure which may have a low positive predictive value, given that some SGA infants are constitutionally small but not growth restricted [61]. We hypothesize that any outcome misclassification is likely to be non-differential, leading to the expectation of bias towards the null.

In addition, although gestational age was available from birth certificates for index pregnancies, for non-index pregnancies, gestational age was reported in categories on the self-administered questionnaire. We used multiple imputation to obtain continuous values of gestational age, including the birth weight and categorical gestational age, in the multiple imputation model. Multiple imputation is a valid method for dealing with missing outcome data and produces less biased results than complete case analysis [62]. In addition, we found that the results were similar when we restricted them to index pregnancies. 

The possibility of non-differential exposure misclassification is high in our study. Our exposure assessment did not take into account the weather, land use, geography, temporal variability, or non-residential sources of exposure to traffic. Monitoring and satellite data were not available for the study years, and we were unable to account for time-activity patterns, given the retrospective nature of the study. However, while traffic proximity metrics have been shown to predict smaller percentages of variability in traffic-related air pollutants than more complex models [63,64], they are precise, easy to measure, and are particularly useful in areas or time periods with limited monitoring data. They also assess the complex mixture of exposures that living close to traffic represents, rather than relying on modeling individual, highly correlated components. In addition, other studies have demonstrated associations between road proximity or density metrics and pregnancy outcomes [16,17,18,35], and one study suggested that road proximity may suffer from less exposure misclassification than more temporally-resolved metrics such as land use regression [22]. 

Our exposure metric lacks temporal resolution; therefore, we were unable to measure the exposure at different time periods throughout the pregnancy and were unable to capture seasonal or annual changes in traffic. The etiologically-relevant window of susceptibility for ischemic placental disease is likely to be the first trimester. We attempted to examine this issue by stratifying our results by season of birth. Traffic on Cape Cod increases dramatically in the summer because it is a popular vacation destination; therefore, births in the spring would have the highest exposure to traffic during the first trimester. We found no evidence of effect measure modification by season of birth; therefore, these data do not support the hypothesis that traffic exposure in the first trimester causes ischemic placental disease.

Individuals who live close to major roadways are more likely to be exposed to other neighborhood-level socioeconomic and environmental factors that could influence the risk of ischemic placental disease [65]. If a strong association exists between any of these factors and the risk of ischemic placental disease, our study results could be affected by unmeasured confounding. Our study population was relatively homogenous; therefore, we did not observe strong expected associations between traffic exposure and socioeconomic variables, and had very little measured confounding. In addition, for an unmeasured confounder to explain our null results, it would have to be positively associated with traffic exposure and negatively associated with ischemic placental disease (or vice versa). We have not hypothesized the existence of any particular variable that meets these criteria; therefore, we believe that unmeasured confounding is unlikely to explain our results.

It is possible that selection bias could explain the largely null findings in this analysis. We excluded early pregnancy losses from our analysis because we cannot ascertain the outcome in these pregnancies. However, there is some evidence that air pollution is associated with an increased risk of early pregnancy loss [66]. By conditioning on pregnancies that survived into the third trimester, selection into the analytic sample may be related to exposure; if early pregnancy loss and ischemic placental disease share a common cause that is not controlled for, our results may be biased downwards. 

Given the small number of pregnancies complicated by preeclampsia, placental abruption, and stillbirth in our study population, our estimates for these conditions were imprecise. In addition, the results for ischemic placental disease were largely driven by the presence of SGA. A larger study population with a greater number of cases could allow for a more precise estimation of the effects of traffic exposure on ischemic placental disease, as well as its individual components. More direct measures of placental function, as have been conducted in other studies [1], could also help elucidate the association and mechanism under study.

## 5. Conclusions

Overall, we found no evidence of an association between exposure to traffic and ischemic placental disease in this retrospective cohort study. The results were relatively consistent within the strata of parity, maternal age, season of birth, and first trimester smoking. We did find some evidence of an increased risk of placental abruption and stillbirth among women living closest to major roadways, but the number of cases was small and the results could have been affected by non-differential misclassification of exposure and selection bias.

## Figures and Tables

**Table 1:** Characteristics of 3309 pregnancies by the occurrence of ischemic placental disease.

Characteristic	Ischemic Placental Disease	
	Yes	No
Number, *n* (%)	270 (8.2)	3039 (91.8)
Year of pregnancy, *n* (%)		
Before 1974	61 (22.6)	570 (18.8)
1975–1980	118 (43.7)	1243 (40.9)
After 1980	91 (33.7)	1226 (40.3)
Maternal age (years), mean (SD)	26.6 (4.5)	27.7 (4.6)
Paternal age (years), mean (SD)	30.1 (6.1)	30.7 (5.8)
White, *n* (%)	256 (94.8)	2956 (97.3)
Maternal education, *n* (%)		
Less than high school	2 (0.7)	39 (1.3)
High school graduate	55 (20.4)	569 (18.7)
Some college	105 (38.9)	1047 (34.5)
Four-year college graduate or more	108 (40.0)	1384 (45.5)
Paternal occupation, *n* (%)		
White collar	114 (42.2)	1521 (50.1)
Blue collar	106 (39.3)	1020 (33.6)
Other	50 (18.5)	498 (16.4)
Number of prior pregnancies and prior IPD, *n* (%)		
Nulligravid	118 (43.7)	905 (29.8)
Previous pregnancy, no prior IPD	122 (45.2)	1962 (64.6)
Previous pregnancy, prior IPD	30 (11.1)	172 (5.7)
Gestational weight gain <20 lb, *n* (%)	70 (25.9)	415 (13.7)
Cigarette smoking during first trimester, *n* (%)	119 (44.1)	744 (24.5)
Alcohol consumption during first trimester, *n* (%)	110 (40.7)	1108 (36.5)
Live <50 m from A1–A3 roadway, *n* (%)	35 (13.0)	443 (14.6)
Live <50 m from A1–A2 roadway, *n* (%)	11 (4.1)	111 (3.7)
Live <50 m from A1 roadway, *n* (%)	0 (0.0)	0 (0.0)
Any A1–A3 roads within 500 m buffer, *n* (%)	237 (87.8)	2650 (87.2)
Any A1–A2 roads within 500 m buffer, *n* (%)	149 (55.2)	1532 (50.4)
Any A1 roads within 500 m buffer, *n* (%)	12 (4.4)	150 (4.9)
Any A1–A3 roads within 200 m buffer, *n* (%)	150 (55.6)	1718 (56.5)
Any A1–A2 roads within 200 m buffer, *n* (%)	117 (43.3)	1086 (35.7)
Any A1 roads within 200 m buffer, *n* (%)	5 (1.9)	39 (1.3)
Any PCE exposure in year prior to pregnancy, *n* (%)	126 (46.7)	1536 (50.5)

IPD = ischemic placental disease; PCE = perchloroethylene; SD = standard deviation.

**Table 2:** Distribution of traffic exposure for pregnancies in the Cape Cod Family Health Study.

	Shortest Euclidean Distance Between Residence and the Closest Major Roadway (m)			Length of Major Roadways within 500 m Buffer Around Residence (m)			Length of Major Roadways within 200 m Buffer Around Residence (m)		
	A1–A3 Roads	A1–A2 Roads	A1 Roads	A1–A3 Roads	A1–A2 Roads	A1 Roads	A1–A3 Roads	A1–A2 Roads	A1 Roads
Minimum	12	13	67	0	0	0	0	0	0
5th percentile	30	68	565	0	0	0	0	0	0
10th percentile	38	136	911	0	0	0	0	0	0
25th percentile	85	339	2075	592	0	0	0	0	0
Median	188	728	3694	1008	16	0	120	0	0
75th percentile	362	1541	5276	1571	658	0	373	94	0
90th percentile	570	2136	7310	2000	1771	0	411	335	0
95th percentile	743	2524	8372	2307	2686	0	555	527	0
Maximum	1923	5306	37,192	3989	11,836	1463	1087	2721	423

**Table 3:** Characteristics of 3309 pregnancies by the shortest Euclidean distance of residence from the closest major (A1–A3) roadways.

	Shortest Euclidean Distance of Residence from Closest A1–A3 Roadway (m)			
	<50	50–99	100–199	≥200
Number, *n* (%)	478 (14.5)	443 (13.4)	877 (26.5)	1511 (45.7)
Year of pregnancy, *n* (%)				
Before 1974	96 (20.1)	108 (24.4)	151 (17.2)	276 (18.3)
1975–1980	216 (45.2)	177 (40.0)	357 (40.7)	611 (40.4)
After 1980	166 (34.7)	158 (35.7)	369 (42.1)	624 (41.3)
Maternal age (years), mean (SD)	27.3 (4.8)	27.6 (4.5)	26.9 (4.4)	28.1 (4.7)
Paternal age (years), mean (SD)	30.2 (6.0)	30.8 (6.0)	29.9 (6.0)	31.2 (5.6)
White, *n* (%)	457 (95.6)	430 (97.1)	850 (96.9)	1475 (97.6)
Maternal education, *n* (%)				
Less than high school	10 (2.1)	2 (0.5)	17 (1.9)	12 (0.8)
High school graduate	72 (15.1)	81 (18.3)	210 (24.0)	261 (17.3)
Some college	181 (37.9)	152 (34.3)	301 (34.3)	518 (34.3)
Four-year college graduate or more	215 (45.0)	208 (47.0)	349 (39.8)	720 (47.7)
Paternal occupation, *n* (%)				
White collar	230 (48.1)	218 (49.2)	395 (45.0)	792 (52.4)
Blue collar	171 (35.8)	165 (37.3)	269 (30.7)	521 (34.5)
Other	77 (16.1)	60 (13.5)	213 (24.3)	198 (13.1)
Number of prior pregnancies and prior IPD, *n* (%)				
Nulligravid	158 (33.1)	130 (29.4)	262 (29.9)	473 (31.3)
Previous pregnancy, no prior IPD	291 (60.9)	287 (64.8)	559 (63.7)	947 (62.7)
Previous pregnancy, prior IPD	29 (6.1)	26 (5.9)	56 (6.4)	91 (6.0)
Gestational weight gain <20 lb, *n* (%)	66 (13.8)	57 (12.9)	122 (13.9)	240 (15.9)
Cigarette smoking during first trimester, *n* (%)	124 (25.9)	116 (26.2)	254 (29.0)	369 (24.4)
Alcohol consumption during first trimester, *n* (%)	161 (33.7)	179 (40.4)	324 (36.9)	554 (36.7)
Any PCE exposure in year prior to pregnancy, *n* (%)	238 (49.8)	221 (49.9)	399 (45.5)	804 (53.2)

**Table 4:** Risk ratios (RR) and 95% CIs for the association between traffic exposure and ischemic placental disease.

Exposure	Events/*N*	Unadjusted RR (95% CI)	Adjusted ^a^ RR (95% CI)
Distance from closest A1–A3 road (m)			
≥200	127/1511	Reference	Reference
100–199	68/877	0.91 (0.66, 1.27)	0.87 (0.63, 1.21)
50–99	40/443	1.11 (0.77, 1.60)	1.08 (0.75, 1.55)
<50	35/478	0.77 (0.49, 1.23)	0.74 (0.47, 1.17)
Length of A1–A3 roads in 500 m buffer (m)			
0	33/422	Reference	Reference
1–1075	124/1443	1.14 (0.74, 1.76)	1.12 (0.73, 1.71)
≥1076	113/1444	1.11 (0.67, 1.85)	1.08 (0.65, 1.77)
Length of A1–A3 roads in 200 m buffer (m)			
0	120/1441	Reference	Reference
1–351	80/933	1.01 (0.73, 1.40)	0.95 (0.69, 1.31)
≥352	70/935	0.88 (0.62, 1.24)	0.85 (0.60, 1.19)

^a^ Adjusted for maternal age at pregnancy (continuous) and year of pregnancy (continuous).

**Table 5:** Risk ratios and 95% CIs for the association between traffic exposure and preeclampsia, placental abruption, SGA, stillbirth, and vaginal bleeding.

Exposure	Preeclampsia		Placental Abruption		SGA		Stillbirth		Vaginal Bleeding	
	Events/*N*	Adjusted ^a^ RR (95% CI)	Events/*N*	Adjusted ^a^ RR (95% CI)	Events/*N*	Adjusted ^a^ RR (95% CI)	Events/*N*	Adjusted ^a^ RR (95% CI)	Events/*N*	Adjusted ^a^ RR (95% CI)
Distance from closest A1–A3 road (m)										
≥200	17/1511	Reference	13/1511	Reference	108/1505	Reference	6/1511	Reference	112/1511	Reference
100–199	9/877	0.89 (0.37, 2.17)	10/877	1.34 (0.54, 3.30)	51/870	0.81 (0.55, 1.19)	7/877	2.02 (0.65, 6.30)	61/877	0.96 (0.68, 1.35)
<100	5/921	0.46 (0.16, 1.29)	13/921	1.75 (0.82, 3.76)	61/915	0.91 (0.63, 1.31)	6/921	1.71 (0.56, 5.23)	42/921	0.67 (0.47, 0.95)
Length of A1–A3 roads in 500 m buffer (m)										
0	5/422	Reference	3/422	Reference	25/420	Reference	2/422	Reference	28/422	Reference
1–1075	14/1443	0.81 (0.29, 2.24)	16/1443	1.57 (0.46, 5.33)	97/1436	1.17 (0.70, 1.95)	7/1443	0.97 (0.21, 4.48)	97/1443	1.08 (0.68, 1.73)
≥1076	12/1444	0.69 (0.23, 2.06)	17/1444	1.67 (0.49, 5.72)	91/1434	1.14 (0.63, 2.05)	10/1444	1.43 (0.33, 6.28)	90/1444	1.02 (0.63, 1.65)
Length of A1–A3 roads in 200 m buffer (m)										
0	16/1441	Reference	12/1441	Reference	96/1435	Reference	6/1441	Reference	107/1441	Reference
1–351	9/933	0.86 (0.35, 2.09)	13/933	1.76 (0.75, 4.15)	60/924	0.86 (0.59, 1.26)	9/933	2.38 (0.81, 7.00)	62/933	0.89 (0.63, 1.26)
≥352	6/935	0.56 (0.21, 1.47)	11/935	1.49 (0.65, 3.38)	57/931	0.86 (0.58, 1.28)	4/935	1.08 (0.31, 3.75)	46/935	0.71 (0.50, 1.01)

^a^ Adjusted for maternal age at pregnancy (continuous) and year of pregnancy (continuous); SGA = small-for-gestational-age.

## Supplementary Materials

The following are available online at www.mdpi.com/1660-4601/14/7/682/s1, Figure S1: Directed acyclic graph showing potential confounders of the relationship between traffic-related air pollution and ischemic placental disease. Variables in italics were not measured in our study.
