# Supplementary material for: Residential Proximity to Roadways and Ischemic Placental Disease in a Cape Cod Family Health Study
Source: Int J Environ Res Public Health. 2017 Jun 24;14(7):682. doi: 10.3390/ijerph14070682 (PMC5551120; doi:10.3390/ijerph14070682)
Supplement: Supplementary file 1 [file ijerph-14-00682-s001.pdf]

Residential Proximity to Roadways and Ischemic Placental Disease in a Cape Cod Family Health Study

Amelia K. Wesselink, Jenny L. Carwile, María Patricia Fabian, Michael R. Winter, Lindsey J. Butler, Shruthi Mahalingaiah and Ann Aschengrau

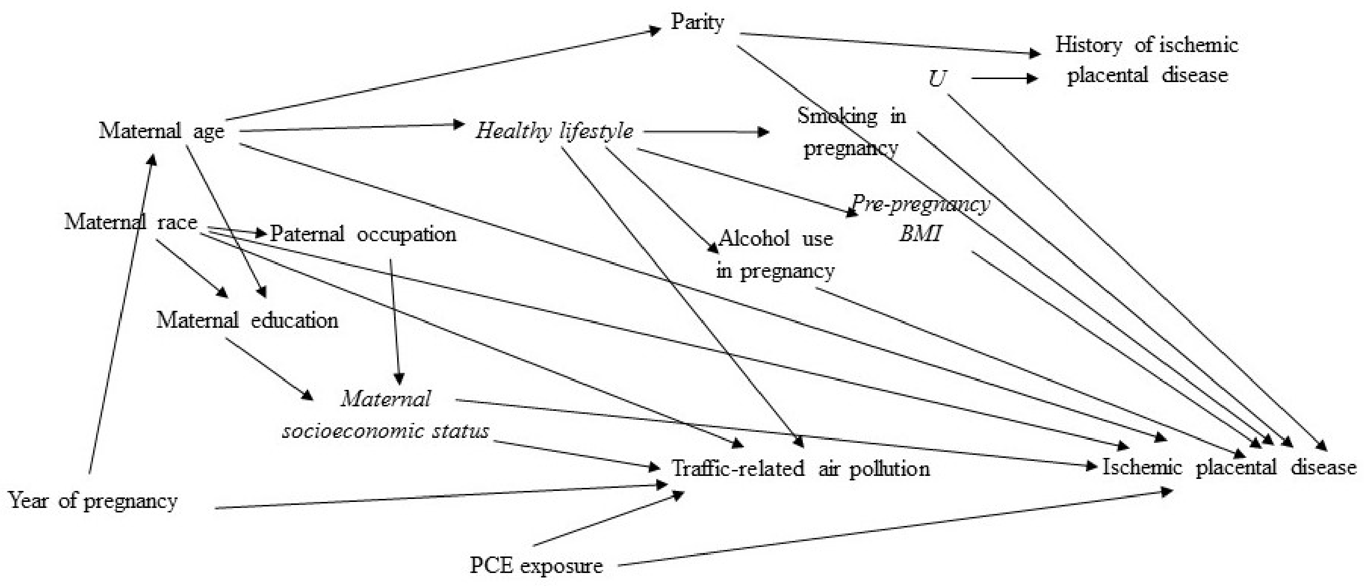

Figure S1. Directed acyclic graph showing potential confounders of the relationship between traffic-related air pollution and ischemic placental disease. Variables in italics were not measured in our study.
